# Evaluating the validity of depression-related stigma measurement among diabetes and hypertension patients receiving depression care in Malawi: A mixed-methods analysis

**DOI:** 10.1371/journal.pgph.0001374

**Published:** 2023-05-17

**Authors:** Josée M. Dussault, Christopher Akiba, Chifundo Zimba, Jullita Malava, Harriet Akello, Melissa Stockton, MacDonald Mbota, Maureen Matewere, Jones Masiye, Michael Udedi, Bradley N. Gaynes, Vivian F. Go, Mina C. Hosseinipour, Brian W. Pence

**Affiliations:** 1 Dept of Epidemiology, UNC Gillings School of Global Public Health, Chapel Hill, NC, United States of America; 2 RTI International, Research Triangle Park, NC, United States of America; 3 UNC Project - Malawi, Lilongwe, Malawi; 4 Malawi Epidemiology and Intervention Research Unit, Karonga, Malawi; 5 Dept of Psychiatry, Columbia University Vagelos College of Physicians and Surgeons, New York, NY, United States of America; 6 New York State Psychiatric Institute, New York, NY, United States of America; 7 Ministry of Health Malawi, NCDs & Mental Health Unit, Lilongwe, Malawi; 8 Dept of Psychiatry, UNC School of Medicine, Chapel Hill, NC, United States of America; 9 Dept of Health Behavior, UNC Gillings School of Global Public Health, Chapel Hill, NC, United States of America; 10 Division of Infectious Disease, UNC School of Medicine, Chapel Hill, NC, United States of America; Transcultural Psychosocial Organization Nepal / KIST Medical College, NEPAL

## Abstract

Mental illness stigma research is sparse in Malawi. Our team previously analyzed the reliability and statistical validity of a quantitative tool to measure depression-related stigma among participants with depressive symptoms using quantitative psychometric methods. This analysis aims to further evaluate the content validity of the stigma tool by comparing participants’ quantitative responses with qualitative data. The SHARP project conducted depression screening and treatment at 10 noncommunicable disease clinics across Malawi from April 2019 through December 2021. Eligible participants were 18–65 years with depressive symptoms indicated by a PHQ-9 score ≥5. Questionnaires at each study timepoint included a vignette-based quantitative stigma instrument with three thematic domains: disclosure carryover (i.e., concerns about disclosure), treatment carryover (i.e., concerns about external stigma because of receiving depression treatment), and negative affect (i.e., negative attitudes about people having depression). Sub-scores were aggregated for each domain, with higher scores indicating greater stigma. To better understand participants’ interpretation of this quantitative stigma questionnaire, we asked a subset of six participants a parallel set of questions in semi-structured qualitative interviews in a method similar to cognitive interviewing. Qualitative responses were linked with participants’ most recent quantitative follow-up interviews using Stata 16 and NVivo software. Participants with lower quantitative stigma disclosure sub-scores had qualitative responses that indicated less stigma around disclosure, while participants with higher quantitative stigma sub-scores had qualitative responses indicating greater stigma. Similarly, in the negative affect and treatment carryover domains, participants had parallel quantitative and qualitative responses. Further, participants identified with the vignette character in their qualitative interviews, and participants spoke about the character’s projected feelings and experiences based on their own lived experiences. The stigma tool was interpreted appropriately by participants, providing strong evidence for the content validity of the quantitative tool to measure these stigma domains.

## Background

Mental illness affects approximately 13% of the global population [1, 2], and approximately 4% of the global population lives with a depressive disorder [1, 2]. Although there are cost-efficient and effective depression treatment options [3, 4], gaps persist in connecting individuals to and maintaining them in depression care. One barrier to engagement in care for patients with depression is the stigma associated with depression. Stigma is defined as a “mark” or discreditable attribute–individuals carrying this attribute are stigmatized, experiencing some reduction or devaluation in their personhood due to the stigma [5, 6]. What is considered to be a discreditable attribute—and the degree to which it discredits—may vary by social context, and tools measuring stigma may therefore need adaptation and validation in specific populations [7].

The bulk of mental illness stigma research has been based in Europe and North America, where studies have demonstrated that patients who report greater mental health stigmatization are also less likely to seek care and more likely to experience discrimination and demoralization [8–11]. Such studies have also demonstrated that mental health stigmatization negatively impacts treatment adherence and response [12, 13]. While research on mental health stigma in Africa is limited, one study in South Africa found that stigma and misinformation regarding mental illness are prevalent and negatively associated with help-seeking behavior [14]. Another study in Blantyre, Malawi characterized community perceptions around mental illness and found that stigma manifested differently in the local context compared to other international studies. For example, participants were simultaneously more likely to attribute the cause of mental illness to God’s punishment or other spiritual causes and also more likely to attribute mental illness to brain disorders [15].

While mental health stigma is an important issue, quantitatively measuring stigma is challenging, particularly in settings where the research is still sparse. To address this issue, we previously conducted a study to quantitatively evaluate the validity and reliability of a depression-related stigma instrument among patients engaged in noncommunicable disease (NCD) care and exhibiting depressive symptoms in Malawi [16]. While quantitative analysis indicated acceptable levels of structural, convergent, and divergent validity and instrument reliability, our study team elected to further assess the content validity of the study instrument by integrating patient qualitative data with the quantitative data already collected [17, 18]. The following study is a mixed-methods analysis of these data.

## Methods

### Ethics statement

This study has been approved by the University of North Carolina Biomedical Institutional Review Board (UNC IRB). It has also been approved by the Malawi National Health Science Research Committee (NHSRC). All study participants provided written informed consent and were provided with a small reimbursement to offset the costs associated with participating in each research interview, as approved by the NHSRC and UNC IRB.

### Study design

The Sub-Saharan Africa Regional Partnership for Mental Health Capacity Building (SHARP) began depression screening in 10 noncommunicable disease (NCD) clinics in Malawi in May 2019, with the overall objective of integrating depression screening and treatment with diabetes and hypertension care at NCD clinics [19]. SHARP participants were recruited using consecutive depression screening as they presented to participating NCD clinics for their standard care. Eligible participants were 18–65 years of age, had elevated depressive symptoms denoted by a score ≥5 on the Patient Health Questionnaire (PHQ-9) [20–24], and had a new or current diagnosis of diabetes or hypertension. SHARP participants completed quantitative interviews at baseline and at 3-month, 6-month, and 12-month follow-up. In December 2020, six SHARP participants who had recently completed 12-month follow-up were selected to complete qualitative interviews as part of the study’s broader qualitative assessment of implementation outcomes. Informed by the study team’s prior experience in collecting formative qualitative data in the same patient population [25], project data collection constraints, and other supporting literature [26–28], six patient interviews were pre-determined to be sufficient to reach saturation *a priori*; this small sample size was particularly supported by the narrow scope of our research topic and the relative homogeneity of the study population [28]. Qualitative interview participants were purposively selected to represent diversity regarding study condition, implementation fidelity, and geographic region [29]. These variables were chosen for the purposive sampling scheme due to the broader implementation-related questions of the larger SHARP study, but the sampling scheme was not expected to dramatically influence patients’ responses to the stigma prompts, which is the scope of the current analysis. We therefore did not anticipate any threat to reaching saturation due to sampling participants across these variables. Interviews were conducted in Chichewa or Chitumbuka via telephone with trained interviewers (MM, MM, and CM) between December 2020 and February 2021. Recorded interviews (average length: 45 min; SD: 11 min) were simultaneously transcribed and translated into English using a one-step approach. The following is an integrated mixed-methods analysis connecting these six participants’ qualitative and quantitative responses to prompts related to stigma. Given the small qualitative sample size and concerns about representativity, we have additionally compared key characteristics of the mixed-methods subsample (n = 6) to the larger sample of quantitative interview participants (n = 781; Table 1).

**Table 1:** Description of sample of SHARP in-depth qualitative interview participants (n = 6) compared to full quantitative sample of SHARP participants who completed 12-month follow-up (N = 781).

	In-depth Interview (IDI) Subsample (n = 6)		Full Sample (N = 781)	
Gender	N	%	N	%
Woman	5	83	634	81
Man	1	17	147	19
**Marital Status (at 12 months)**				
Never Married	0	0	17	2
Married	3	50	526	67
Widowed	2	33	127	16
Divorced/Separated	1	17	111	14
**Employment Status**				
Employed	5	83	601	77
Unemployed	1	17	180	23
	**Mean**	**SD**	**Mean**	**SD**
**Age**	47.5	9.9	50.9	9.7
**Time between 12-month interview and IDI (days)**	125.7	54.4	N/A	N/A
**Baseline PHQ-9 Score**	8.9	2.7	8.1	4.5
**12-month PHQ-9 Score**	3.5	3.7	4.5	3.7
**Stigma Scales**				
Treatment (baseline)	1.5	1.0	1.4	1.1
Disclosure (baseline)	2	1.3	2.4	1.1
Negative affect (baseline)	1.8	0.7	2.2	1
Treatment (12-month)	0.6	0.5	1.1	0.9
Disclosure (12-month)	1.9	1.6	2.2	1.2
Negative Affect (12-month)	0.8	0.7	1.8	1

### Quantitative measurement tool for depression-related stigma

The outcome of interest, patients’ levels of depression-related stigma, was measured using a brief 9-item instrument that was adapted from the Stigma in Global Context–Mental Health Study (SGC-MHS) [7, 30]. The SHARP team adapted this instrument to the social and linguistic context of the SHARP study, identifying key prompts to include and translating them to Chichewa and Chitumbuka. Prior statistical analyses of the measure’s validity and reliability have demonstrated that the stigma questions group around three domains: negative feelings or attitudes toward individuals with depression (negative affect), the role of disclosure particularly on the family (disclosure carryover), and social isolation as a result of engaging in treatment (treatment carryover) [16, 31, 32]. Disclosure carryover prompts were centered around the family due to the importance of family in this population and the indication from previous research that stigma spills over onto the family [15, 33]. Team members who translated the stigma instrument were fluent in the respective target language.

**Fig 1:**
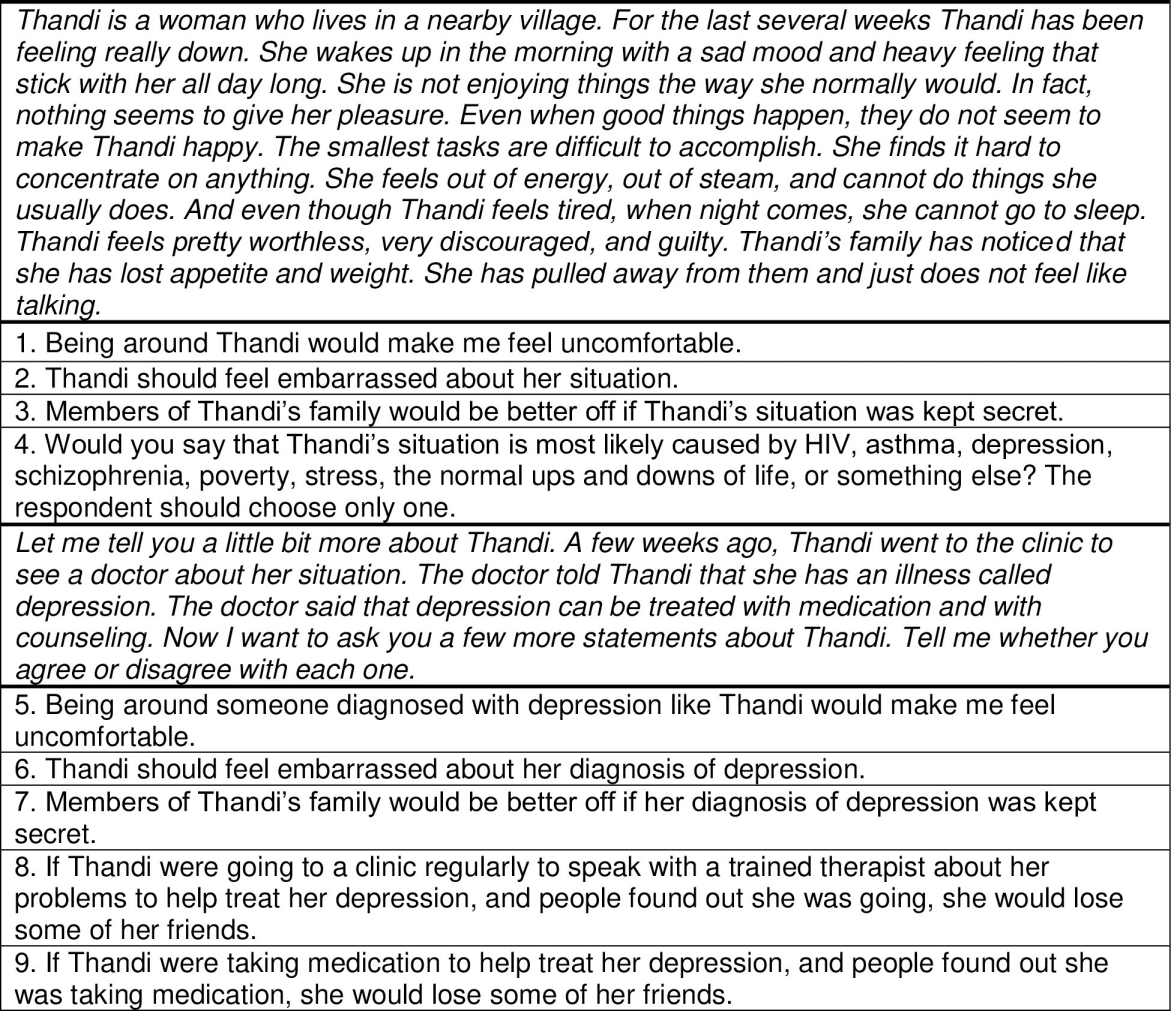
Quantitative stigma questionnaire items.

The stigma instrument first introduced a vignette of a woman named Thandi and described her depressive symptoms without naming it as depression. Participants then rated their level of agreement with statements about whether Thandi’s situation was shameful or embarrassing, for instance, in three prompts on a 5-point Likert scale (Fig 1). A fourth prompt then asked participants to select from a list of options what they believed to be the cause of Thandi’s situation. A second segment of the vignette then explained that Thandi had been diagnosed with depression and presented five more prompts, closely parallel to the first set of prompts, on the same 5-point Likert scale. Each prompt was written such that agreement endorsed depression-related stigma. Strong agreement with any given prompt was equivalent to 4 points, while strong disagreement was 0 points. All SHARP participants completed the quantitative stigma assessment as part of the quantitative interview at each SHARP study timepoint.

### Qualitative interview guide: Stigmatizing beliefs and experiences

To better understand participants’ thought processes when responding to the quantitative stigma instrument, we included a series of parallel, open-ended questions in a semi-structured interview guide (S1 Appendix). Prompts were presented while using an identical vignette to that of the quantitative stigma tool, this time using a character named Mary. Subsequent questions asked participants directly about their own experiences of stigma before, during, and after participation in the SHARP study. The semi-structured guide was revised by study team members using an iterative process before being translated from English to the target languages. Because it was used for qualitative interviewing, semi-structured interview guides did not require prior validation. However, interviewers underwent thorough training with the study team before collecting participant interviews to ensure the questions and prompts captured the information that the study team sought.

### Mixed-methods analysis methods

This mixed-methods analysis used complementary quantitative and qualitative data, with qualitative data collected after quantitative data (quan > qual approach), to confirm the validity of the quantitative instrument used in the SHARP study [17, 34–38]. We used a convergent triangulation design to simultaneously evaluate the quantitative and qualitative data [38, 39]. Using this triangulation design also helps bolster the weaknesses of one method (e.g., lack of depth in quantitative analysis) with the strengths of the other (e.g., depth of content in qualitative analysis) [37, 38]. Qualitative interview transcripts were linked with participants’ quantitative surveys at baseline and 12-month follow-up using Stata 16.1 and NVivo (released in March 2020) [40, 41].

Based on prior exploratory factor analysis [16], the authors hypothesized that qualitative interview themes would also cluster around topics of disclosure carryover, treatment carryover, and negative affect toward individuals exhibiting depression symptoms. Therefore, using theoretical thematic analysis, these themes of interest were included in the “start list” of codes, which was generated prior to reviewing participants’ transcripts [42, 43]. Then, two team members (JD, CA) reviewed all six transcripts and met to revise the codebook, adding codes that highlighted phenomena that arose organically in the interviews with a general inductive approach [44]. A third revision of the codebook further refined codes for qualitative analysis after pilot coding (S2 Appendix). Two team members (JD, CA) then co-coded two transcripts to test the robustness of the final codebook before one team member (JD) ultimately coded all six transcripts using theoretical thematic analysis [43, 45]. The coders and interviewers maintained open communication as an additional form of peer debriefing [46, 47].

Throughout the coding process, the primary coder (JD) annotated transcripts to summarize cases and draw comparison to other cases using a constant comparative method [45, 48]. Upon completing coding, participants’ quantitative survey data were linked as part of their case attributes in NVivo [41]. Cross-case displays were used to compare annotations, codes, and case attributes between participants [42, 49]. Case-ordered descriptive meta-matrices and two-variable case-ordered matrices were the most common forms of cross-case displays used as they allowed participants’ open-ended responses and quantitative characteristics to be displayed simultaneously [42, 49]. Excerpts of participant responses and attributes are presented around specific themes and patterns. De-identified matrices can be found in supplementary material (S1 and S2 Tables).

## Results

Study results are organized in six sub-sections: participant characteristics, thought processes in interpreting the stigma measure, three key domains that the stigma tool was expected to measure (treatment carryover, disclosure carryover, and negative affect), and participants’ understanding of depression as an illness.

### Participant characteristics

The six qualitative participants initiated the program between May and November 2019 and completed 12-month follow-up between June and November 2020. The mean time between quantitative data collection and qualitative interview was 126 days (SD: 54 days). Five participants were female, and one was male. While small, this subsample demonstrated similar distributions of most characteristics compared to the full sample of 781 participants (Table 1).

### Process: Identifying with the vignette character

Participants were first primed with the vignette before being asked about their own experiences of stigma related to depression diagnosis or treatment. Generally, participants’ responses about their own experiences mirrored the anticipated experiences that they projected onto the vignette character. Moreover, many participants expressly stated their identification with the vignette. For example, one participant compares her own situation to Mary’s by referring to herself as a “living example” of what is possible with treatment:

It can affect Mary in a positive way because, if Mary is treated, she will lead a healthy life and her family members can be happy…I am thinking in this way because I am a living example. When I was diagnosed with this problem, I was very miserable. When I was depressed, I never wanted people to know about my problem but after counseling I started living a normal life.

Similarly, another participant framed her depression experiences in relation to Mary’s by saying that she was like Mary, “this girl from [her] community”, before echoing the depressive symptoms that were described in the vignette: “I was concerned during this time because I was like this girl from my community. I also did not want to be seen by any other person. I wanted to isolate myself and cry…Food and everything…was never interesting.”

This pattern of identifying with the vignette was also recognizable in the qualitative interview with a male participant, suggesting that the gender of the vignette character did not prevent the male participant from relating to her story. When probed about why he believed that he could be comfortable around Mary, the participant stated the following:

Because she is also human, and every human being has a right to receive care from another person. In the same way when I was ill, …For me to be in this condition where I am discussing with you now, it shows you that a huge effort was made. I know I could have died. But they told me, counseled me gradually and patiently, so that is why I am now fine.

All participants recognized the depressive symptoms that were described in the first vignette and used the words “depressed” or “depression” before being told, in the second portion of the vignette, that Mary had been diagnosed with depression by the doctor. This recognition of the symptoms appears to have aided the participants in identifying with the vignette character. Likewise, in the quantitative 12-month survey, five of the six participants correctly named the vignette character’s condition as depression, and just one named it as “stress”, which is often a proxy term used for depressive symptoms in Malawi [16, 50].

### Theme 1: Treatment carryover

Participants’ quantitative responses in the treatment carryover domain—which focused on losing friends due to the stigma of receiving treatment—trended downward from baseline to 12-month follow-up. In other words, after 12 months in the study, participants expressed greater disagreement with prompts stating that Thandi would lose friends as a result of seeking treatment for depression. At 12-month follow-up, the qualitative sub-sample had treatment subscale scores ranging from 0 (strong disagreement) to 1 (disagreement). Participants with scores greater than 0 had more references to stigma due to treatment seeking in their interviews compared to participants with scores of 0. There was only one participant with a subscale score of 0 who made a reference to treatment carryover, and that reference was overall quite positive: “Even for me, if my friends would know that I am receiving medication for depression, I do not see any problem.”

Participants with treatment carryover subscale scores of 0.5 or 1 also had generally positive experiences with sharing information about their treatment: “One of [my] children … asked me, ‘What will you do at the hospital?’, so I told him. Then he said, ‘Mum, that is good news! …Please be adherent to this study.’” Overall, the qualitative interview participants had positive experiences with sharing information about their treatment. Nevertheless, participants with treatment carryover scores greater than 0 did have more detailed descriptions of the treatment-seeking stigma they had anticipated when they were first diagnosed with depression. For example, the following excerpt describes one participant’s initial fear of losing friends. This participant had a treatment carryover score of 2 (neutral) at baseline:

**Interviewer (I):** So, when you started receiving care for depression, what concern did you have that your friends may know?

**Participant (P):** I thought that my friends would discriminate against me because they would think I don’t think properly…It affected me in a negative way because I was receiving the counseling in order to find solutions to my problems but at the same time if people knew that I am receiving counseling, they would think I fail to make decisions on my own. Because some people are counseled because they fail to make their own decisions for their lives…It affected me because sometimes I thought, “Am I going to complete my counseling sessions?” Sometimes I had these opinions, and I was feeling that “How are my friends regarding me?”

**I:** How did those concerns change the way you received the care?

**P:** Okay my concern changed because I regarded my life as vital. I never minded what people would say because my life was more important than what they were saying, and I ended up living a happy life and even my children are happy…The best person I salute is the one who counseled me because she told me about the importance of a healthy life, and what makes a person live a happy life? Even if people knew about my confidential issues, it should not be an issue to me because everyone is responsible for her own life.

This participant’s excerpt keenly describes the experiences of individuals who anticipated higher levels of stigma around treatment carryover at the time of diagnosis and who may have struggled with attending their required appointments as a result of such stigma. Similarly, another participant (12-month treatment carryover score: 0.5, baseline score: 2), speaking of the vignette character, described other risks of frequent clinic visits: “Some of her friends can feel happy because they would know that Mary is going to be assisted—That is if they knew what depression is all about; but if they don’t know, they may think that Mary has a certain condition that is contagious and that it may also affect them.” This perspective again could reduce the frequency with which some patients may decide to attend clinic or otherwise influence their treatment plan and should be seriously considered when establishing treatment with patients.

### Theme 2: Disclosure carryover

Of the three stigma subscales, the disclosure carryover subscale had the highest scores at 12-month follow-up, with a range from 0 to 4. Participants with the least stigmatizing quantitative scores believed that Mary would benefit from disclosing her condition because it would allow her family and friends to provide greater support. They often provided nuanced perspectives, recognizing that disclosure could present risks, but they still overwhelmingly believed that disclosure would be beneficial for Mary and her family. An excerpt from an interview with a participant whose 12-month follow-up score was 0.5 demonstrates this:

**P:** If she discloses her problem to the family members, it will help them to think about ways to deal with the problem and this will assist in eliminating the problem.

**I:** Okay… [let us consider] that this issue has been known by other people: how would her family members be affected?

**P:** Okay, on that one, it depends on how the family members perceive it because if those other people may know, maybe they can assist her, and she can feel happy, because sometimes we are free to disclose our issues to other people rather than family members.

This participant, while enthusiastically supporting disclosure, was also aware that there could be some spillover effects, or family may not always be as supportive as other members of one’s social network. Similarly, another participant with a score of 0 at 12-month follow-up also provided perspective on disclosing their depression diagnosis: “The other thing that I want to add is that people are different. Other people would say, ‘People will laugh at me if I disclose!’ But this is not the way it is…When you disclose your illness or your innermost thoughts, you will feel free because people will assist you… So, the problem would be manageable.” Overall, participants with low disclosure carryover scores understood the dilemma around disclosure, but they saw more positives than negatives, and they individually had positive experiences—like receiving necessary social support—when disclosing their depression to people.

Participants with the highest subscale scores generally had parallel qualitative responses about whether Mary’s situation should be kept secret. For example, one participant who maintained a subscale score of 4 at baseline and at 12-month follow-up described the risk of Mary’s symptoms worsening if her condition were public knowledge in her community:

**I:** Apart from her getting counseling, should Mary keep secret her situation from her neighbors?

**P:** Yes…I was thinking that she should keep this as a secret because if she shares it with other people, it will be something that will still bring humiliation to Mary…If her situation is not kept a secret, Mary will keep on being depressed for a long time.

This same participant had previously described the stigmatization they had experienced from neighbors due to the physical manifestation of their depressive symptoms, in combination with their diabetes and hypertension diagnoses: “I was not feeling well. I was always weak and while at home, people were not speaking kindly to me, saying, ‘You have lost weight…’ many things to that effect… [People were saying], ‘She is diabetic, and her life is almost over… she is just waiting for the day she dies.’” Living in such a community appears to have influenced the participant’s interpretation of Mary’s disclosure risks such that the participant thoroughly believed Mary was better off keeping her depression a secret.

### Theme 3: Negative affect

Similar to the treatment carryover subscale, participants generally disagreed with statements of negative affect toward the vignette character in their 12-month quantitative surveys, with scores ranging from 0 to 1.5. In qualitative interviews, participants with subscale scores of 0 made it clear that they did not believe that Mary should feel embarrassed or ashamed, e.g., “Mary should not feel embarrassed because of her situation.” Nevertheless, they could understand why she might have such feelings: “Yes, the patient might think [that their depression is shameful], however, once they are counseled, they will feel it is not so. And they will feel better, and they would not be ashamed.” Participants with subscale scores of 0 unilaterally stated that they would not be uncomfortable around Mary, because they recognized her symptoms as depression and felt she needed social support: “It would not be a problem for me to be near Mary, because I have realized that having depression or anxiety is an illness, so … I would sit with her and advise, counsel and reassure her that she should not be distressed or anxious.”

Participants who described feelings of discomfort toward Mary appeared concerned about Mary’s depressive symptoms and well-being. One example from a participant with a 12-month subscale score of 1 and baseline score of 2.75 demonstrates such concerns: “When you have a friend whom you have been chatting with in the past but has shown that she is depressed, you start thinking that her life is at risk, she can develop a health problem that can cost her life. So, you start thinking about it: ‘I will lose my friend.’” Participants’ discomfort with Mary was largely due to her depressive symptoms and concern for whether she was going to seek treatment. They described how it might be uncomfortable to be around someone who is exhibiting depressive symptoms, while still focusing on their concern for the well-being of the individual experiencing a depressive episode: “It cannot make me feel comfortable …she is just staying in the house, depressed, not able to eat… How is she going to live like that?” Overall, participants demonstrated an understanding of depression that moderated their interpretation of Mary’s behavior or diagnosis as shameful or embarrassing, and only some participants found Mary’s behavioral symptoms of depression to be potentially discomforting. However, participants who suggested that Mary’s depressive symptoms would make them uncomfortable were still not uncomfortable by her depression diagnosis, exemplified by all six of the participants’ disagreement with the 12-month quantitative survey prompt, “Being around someone diagnosed with depression like Thandi would make me feel uncomfortable.” By contrast, two of the six participants agreed with the first prompt offered after the first vignette: “Being around Thandi would make me feel uncomfortable.” This first vignette only described her symptoms, which was what participants reacted the most negatively to.

### Theme 4: Depression as an illness

One important theme arose organically in qualitative interviews: the conceptualization of depression as an illness. Participants with more references to depression being an illness in their qualitative interviews tended to have lower subscale scores in the treatment carryover and negative affect domains. However, this trend was not observed among disclosure carryover scores, which were variable among participants who more frequently referenced depression as an illness.

Participants also described how their understanding of depression as an illness emerged when they were first diagnosed with depression, which typically occurred during the clinical visit when they were screened and enrolled in the SHARP study. Participants explained how identifying their collection of depressive symptoms as a medical illness helped them. As one patient describes, “There was an additional thing because it helped me realize—‘Are the symptoms that I have been experiencing related to depression? Am I in the group of those people who are depressed?’ So, it was during this visit when I realized that I have depression as an illness.” A second patient described this phenomenon in the following way: “The other thing about how my life was affected on this first day, is like what I already explained: I did not know anything to say that depression is a disease. So, when I got to know about it, I was really concerned that depression or anxiety is an illness.” Participants recognized the gravity of depression, and through diagnosing these symptoms as depression, participants seem to be highly amenable to treatment. A third patient demonstrates this: “She [Mary] is not supposed to feel ashamed. I feel that if she can receive treatment for this problem, she can be assisted.” The emphasis on treatment, particularly counseling, as necessary to addressing the depressive symptoms described for the vignette character was quite salient across participant interviews. Recognizing depressive symptoms and understanding depression as an illness—rather than a character flaw—were likely important steps in treatment acceptance and recommendation.

## Discussion

This integrated mixed-methods analysis aimed to assess the content validity of a short quantitative stigma questionnaire among patients who received a depression diagnosis in Malawi using additional qualitative data from a subsample of six participants. On average, participants’ quantitative stigma scores decreased between their baseline interview and their 12-month quantitative interview, and qualitative interviews demonstrated that participants’ thinking about depression and stigma had changed since the initiation of their treatment. Further, participants indicated that their understanding of depression as an illness began with treatment initiation, and their understanding of depression as a medical condition was associated with reduced stigma in qualitative and quantitative assessments within the domains of treatment carryover and negative affect. These observations are consistent with prior research suggesting that patients who understand depression to be a medical condition are more likely to be amenable to medical treatment [25, 51, 52]. Understanding depression as an illness was not associated with disclosure scores, which were more often driven by patients’ direct experiences with disclosure and social support. Understanding depression as an illness is particularly important among Malawian patients, where other public beliefs may attribute mental health disorders to alcohol use, drug use, or spirit possession [15, 53].

The process by which participants identified with the vignette character also supports the validation of the quantitative stigma instrument. Previous research establishes that when vignettes closely align with the experience of participants, their responses are related to their own lived experiences or their familiarity with others’ experiences in these scenarios [54, 55]. Thus, the finding in this analysis that participants spoke about the hypothetical experiences of Mary, the vignette character, based on their own experiences aligns with other research using vignettes. Given that participants’ quantitative responses closely matched their qualitative responses and sentiments, it follows that the participants responded to the quantitative instrument—which used the same vignette with the character named Thandi—based on their own anticipated or lived experiences. Further, the male participant in this study still identified with the female vignette character, supporting the assumption that the vignette character’s gender did not bias the responses of male participants in the quantitative survey.

Key limitations include the relatively small sample size of qualitative interview participants, which represents a threat to reaching meaning saturation [56]. Nonetheless, this analysis reached code saturation within the scope of the narrowly-defined themes that we presented here, likely because the patient population was relatively homogenous and the study question was highly specific [27, 28, 56]. Another limitation of this study may be selection bias: this was a subsample of participants who completed 12 months of follow-up for a study related to depression, and participants with characteristics that may have precluded them from completing the study were then likely excluded from this subsample, reducing the generalizability of results. However, it is worth noting that, among participants that were eligible for their 12-month interview prior to the conclusion of the SHARP study’s data collection phase, over 90% successfully completed the 12-month interview. Beyond retention, other study strengths include the breadth of data available in this cohort and the use of qualitative methods to cross-check the content validity of this quantitative instrument. A mixed-methods approach has uniquely allowed us to integrate information and assess the content validity of the stigma tool, and it is our hope that such methods continue to expand in the field of psychometrics [17, 36].

Overall, this study further supports the validity of this brief depression-related stigma instrument. Among individuals living with depression in Malawi, anticipated and experienced stigma present difficulties that may inhibit their recovery. Tools to measure stigma—like the one used in this study—are essential to better identify stigmatizing beliefs that are held among stigmatized individuals and thereby better understand the unique barriers that these individuals face. This abbreviated tool has demonstrated several forms of validity and reliability and may be recommended for use in similar patient populations [16]. Nevertheless, applications of this tool in certain study populations, e.g., among patients living with both depression and HIV, may not yet be advisable without further validation.

## Supporting information

S1 Appendix Patient interview guide. (PDF)

S2 Appendix Analysis codebook with code definitions. The bold, left-aligned topics are the parent codes, and the indented plain-text topics represent sub-codes belonging to the most recent parent code.
(PDF)

S1 Table Number of coding references by relevant patient attributes. One row per thematic code.
(XLSX)

S2 Table Number of words coded within a code by relevant patient attributes. One row per thematic code.
(XLSX)
