# Supplementary material for: Evaluating the validity of depression-related stigma measurement among diabetes and hypertension patients receiving depression care in Malawi: A mixed-methods analysis
Source: PLOS Glob Public Health. 2023 May 17;3(5):e0001374. doi: 10.1371/journal.pgph.0001374 (PMC10191271; doi:10.1371/journal.pgph.0001374)
Supplement: S1 Appendix — (PDF) [file pgph.0001374.s001.pdf]

Interviewer Name: _______________________________ Participant ID: ________________________
Date of Interview: ___ (DD) / ___ (MM) / ______ (YYYY) Start time: ___:___ End time: ___:___

**SHARP Patient Qualitative Guide**

**v2.2 (5th October 2020)**

Hello, my name is [INTERVIEWER NAME], and I am working with researchers at UNC-Project in Lilongwe. We are conducting interviews with individuals who have been participating in the integration of depression services and NCD care. We are asking for your feedback so we can make improvements to the intervention for future use. No intervention or individual is perfect; please do not worry about hurting our feelings or the feelings of those you work with – I can ensure your information and responses will be kept confidential. If you found that something was not to your liking, please let us know so we can make the intervention better for NCD patients across Malawi.

As a reminder, you are not required to answer my questions, and you may skip any questions that make you uncomfortable. If you decide that you no longer want to participate in this interview, it will not affect anything regarding you status at this hospital or on our study. As a reminder, I will use a digital recorder to record our conversation.

Do you have any questions before we begin the interview?

**TURN ON DIGITAL RECORDER**

**Begin by stating: I am [INTERVIEWER NAME] interviewing participant [PARTICIPANT #] on [DATE] [START TIME].**

| **Topics and Main Questions** | **Probes** |
|---|---|
| **(1) Warm-up Questions** | |
| How long have you been coming to this clinic for NCD services?<br><br>How have you liked the services provided by the NCD clinic overall?<br><br>What can you tell me about depression?<br><br>What are the best ways to treat depression? | |
| **(2) ABCD+FB Fidelity** | |
| If possible, could you please tell me everything you can remember about the first clinic visit in which depression was discussed? (Quality) | How did this visit make you feel? (Participant responsiveness) |
| Have you ever taken medication for depression? | If so, how have you felt about the medication? |

| | |
|---|---|
| Have you ever met with a Friendship Bench counselor? | If so, how have your meetings with them been overall? (Participant responsiveness)<br><br>What sorts of things have they spoken to you about? (Content) |

| **(3) Stigma – Vignette/General** | |
|---|---|
| Now I am going to describe a person to you, and then ask you a few questions about that person's situation.<br><br>*Mary is a woman who lives in a nearby village. For the last several weeks, Mary has been feeling really down. She wakes up in the morning with a sad mood and heavy feeling that stick with her all day long. She is not enjoying things the way she normally would. In fact, nothing seems to give her pleasure. Even when good things happen, they do not seem to make Mary happy. The smallest tasks are difficult to accomplish. She finds it hard to concentrate on anything. She feels out of energy, out of steam, and cannot do things she usually does. And even though Mary feels tired, when night comes, she cannot go to sleep. Mary feels pretty worthless, very discouraged, and guilty. Mary's family has noticed that she has lost appetite and weight. She has pulled away from them and just does not feel like talking.*<br><br>Now I want to ask you a few questions about Mary's situation. | |
| How would you feel about Mary? | How comfortable would you feel around her?<br><br>Can you tell me more about that? |
| How do you think Mary would feel about her situation? | How much embarrassment, if any, do you think she should feel?<br>Can you tell me more about that? |
| How secretive, if at all, do you think Mary should be about her situation? | If she did not keep her situation secret, how would this affect Mary's family?<br><br>If Mary did not keep her situation secret, what might happen to her friendships? |
| *Let me tell you a little bit more about Mary. A few weeks ago, Mary went to the clinic to see a doctor about her situation. The doctor told Mary that she has an illness called depression. The doctor said that depression can be treated with medication and with counseling. Now I want to ask you a few more statements about Mary.* | |

| | |
|---|---|
| How would you feel about being around someone diagnosed with depression like Mary? | How comfortable would you feel around her?<br><br>Can you tell me more about that? |
| How do you think Mary would feel about her depression diagnosis? | How much embarrassment, if any, do you think she should feel?<br>Can you tell me more about that? |
| How secretive, if at all, do you think Mary should be about her diagonsis? | If she did not keep her situation secret, how would this affect Mary's family? |
| What if people found out that Mary was receiving counseling at a clinic to treat her depression? How would people react? | What might happen to her friendships? |
| What if people found out that Mary was receiving medication to treat her depression? How would people react? | What might happen to her friendships? |

| **(4) Stigma - Personal** | |
|---|---|
| Now I want to ask you a few questions about your experiences with depression. | |
| At the time you started depression treatment, what bothered you most? | |
| When you began depression treatment, how concerned were you about your friends knowing? | To what extent did it affect your treatment?<br><br>How did your concern for them knowing change during your treatment? |
| When you began depression treatment, how concerned were you about your family knowing? | To what extent did it affect your treatment?<br><br>How did your concern for them knowing change during your treatment? |

| | |
|---|---|
| How much did you share with your friends that you were receiving depression treatment? | Could you say more about why you chose to share that amount?<br><br>To what extent did you describe the treatment to them? How did they respond? |
| How much did you share with your family that you were receiving depression treatment? | Could you say more about why you chose to share that amount?<br><br>To what extent did you describe the treatment to them? How did they respond? |

| **(5) Acceptability** | |
|---|---|
| What costs did you incur related to your depression treatment at the NCD clinic? | How do you feel about these costs?<br><br>What could be done to change how you feel about these costs? |
| How has your depression changed (if at all) since being treated at this clinic? | What do you think has caused the change in your depression? |
| **(6) Conclusion** | |
| What else have we not yet discussed that you think is important for the research team to know about? | |
| I want to thank you for your time today and for participating in our study. Your answers to our questions help us understand how to improve this program for the future. | |

**End by stating: I am [INTERVIEWER NAME] interviewing participant [PARTICIPANT #] on [DATE] [END TIME]**

**TURN OFF DIGITAL RECORDER**
