# Supplementary material for: Evaluating the validity of depression-related stigma measurement among diabetes and hypertension patients receiving depression care in Malawi: A mixed-methods analysis
Source: PLOS Glob Public Health. 2023 May 17;3(5):e0001374. doi: 10.1371/journal.pgph.0001374 (PMC10191271; doi:10.1371/journal.pgph.0001374)
Supplement: S2 Appendix — The bold, left-aligned topics are the parent codes, and the indented plain-text topics represent sub-codes belonging to the most recent parent code. (PDF) [file pgph.0001374.s002.pdf]

S2 Appendix. Analysis codebook with code definitions. The bold, left-aligned topics are the parent codes, and the indented plain-text topics represent sub-codes belonging to the most recent parent code.

Name	Description
**Depressive Symptoms**	**Patient describes their depression severity or their depressive symptoms at any time.**
After Starting Treatment	Patient describes severity of depressive symptoms **after** beginning counselling and/or medication
Prior to Treatment	Patient describes severity of depressive symptoms **prior to** beginning counselling and/or medication.
**Depression Diagnosis**	**Patient describes when they first learned that they had depression.**
Disclosure	Patient describes anything related to disclosing their diagnosis of depression to others.
Identifying depression	Patient explains anything about depressive symptoms being hard to recognize or people in the community not being familiar with depression as a health condition.
**Patient Stigma**	**Blanket code for any discussion of stigma/discrimination from the patient's perspective (excluding the vignette character).**
Stigma due to depressive symptoms	Patient describes having experienced or anticipated stigma due to their depressive symptoms. The absence of this form of stigma, where expected, would still be highlighted with this code. Where applicable, this code will often be co-coded with other "patient stigma" sub-codes.
Stigma due to diagnosis	Patient describes having experienced or anticipated stigma due to their diagnosis of depression. The absence of this form of stigma, where expected, would still be highlighted with this code. Where applicable, this code will often be co-coded with other "patient stigma" sub-codes.
Stigma due to treatment seeking	Patient describes having experienced or anticipated stigma due to their treatment seeking behaviors (i.e., attending clinic a lot). The absence of this form of stigma, where expected, would still be highlighted with this code. Where applicable, this code will often be co-coded with other "patient stigma" sub-codes.
Anticipated Stigma	Stigma anticipated at baseline; described by patient in retrospect. The absence of this form of stigma, where expected based on the question, would still be highlighted with this code.
Experienced Stigma	Descriptions of stigma that the patient experienced at any time. The absence of this form of stigma, where expected, would still be highlighted with this code.
Internalized Stigma	Patient describes their internal thoughts about having depression, or they describe the way that stigma has affected the way they see themselves. This code may sometimes be interpretive based on what they say about themselves/thought about themselves.
Depression as an Illness	Patient displays a biomedical understanding of depression; they describe depression as an illness or use language that shows that depression requires treatment or can lead to death (when not treated)
**Social Support**	**Patient describes forms of social support that they have experienced (or lack). When patient is describing the loss or absence of important/supportive people in their life (often due to their death), use this code.**
**Vignette Character**	**This code describes the part of the interview where the interviewer and respondent are discussing the vignette about Mary.**
Able to Recognize Symptoms as Depression	Patient identifies depressive symptoms as depression prior to the second vignette where they are told that she was diagnosed with depression.
Identifying with Vignette Character	This is a process code to identify the degree to which participants describe themselves and their experiences in relation to the vignette character's story.
Social Support	Patient describes social support that they would expect for the vignette character to have, or support that they themselves would offer the vignette character.
Disclosure	Discussion of whether Mary should disclose and/or ownership of health information & privacy.
Stigma or Discrimination	Patient describes stigma or discrimination (of any form, internal or external) that they would expect for the vignette character to encounter. Discussions of shame are pooled into this category. The absence of stigma, where expected, would still be highlighted with this code.
